# Chloral in Cholera Morbus

**Published:** 1874-01

**Authors:** Louis B. Bouchelle

**Affiliations:** Midville, Burke County, Georgia


CHLORAL IN CHOLERA MORBUS.—For two years I have used this drug in cholera morbus with marked succcss. It may seem strange, but it is no more strange than true, that a single dose for adults, of twenty to thirty grains, has in every instance served to quiet the stomach and bowels, and the patient drops into a refreshing slumber from which he wakes in a few hours, *well*, except some weakness and soreness. Such has been the result with patients in the prime of life, and with the aged and infirm generally. Try it.

LOUIS B. BOUCHELLE.

MIDVILLE, BURKE COUNTY, GEORGIA.

